# Non-target-Site Resistance in *Lolium* spp. Globally: A Review

**DOI:** 10.3389/fpls.2020.609209

**Published:** 2021-01-22

**Authors:** Andréia K. Suzukawa, Lucas K. Bobadilla, Carol Mallory-Smith, Caio A. C. G. Brunharo

**Affiliations:** ^1^Department of Crop and Soil Science, Oregon State University, Corvallis, OR, United States; ^2^Department of Crop Sciences, University of Illinois, Urbana, IL, United States

**Keywords:** altered herbicide translocation, herbicide metabolism, multiple-herbicide resistance, cross-herbicide resistance, ryegrass, *Lolium rigidum* Gaud, *Lolium multiflorum* (Lam.), *Lolium perenne* (L.)

## Abstract

The *Lolium* genus encompasses many species that colonize a variety of disturbed and non-disturbed environments. *Lolium perenne* L. spp. *perenne*, *L*. *perenne* L. spp. *multiflorum*, and *L*. *rigidum* are of particular interest to weed scientists because of their ability to thrive in agricultural and non-agricultural areas. Herbicides are the main tool to control these weeds; however, *Lolium* spp. populations have evolved multiple- and cross-resistance to at least 14 herbicide mechanisms of action in more than 21 countries, with reports of multiple herbicide resistance to at least seven mechanisms of action in a single population. In this review, we summarize what is currently known about non-target-site resistance in *Lolium* spp. to acetyl CoA carboxylase, acetohydroxyacid synthase, microtubule assembly, photosystem II, 5-enolpyruvylshikimate-3-phosphate synthase, glutamine synthetase, very-long chain fatty acids, and photosystem I inhibitors. We suggest research topics that need to be addressed, as well as strategies to further our knowledge and uncover the mechanisms of non-target-site resistance in *Lolium* spp.

## Introduction

The *Lolium* genus contains many species of economic importance. *L. perenne* L. ssp. *perenne* (*L. perenne)*, *L. perenne* L. spp. *multiflorum* (*L. multiflorum*), and *L. rigidum* are of particular relevance due to their widespread presence globally. These three species (hereinafter referred collectively to as *Lolium* spp.) are diploid (*2n* = *2x* = 14), obligate outcrossing, and interfertile grass species that are widely planted for cover crop, turf, and pasture. These species are also considered weeds of agricultural and non-agricultural areas, and exhibit a distinctive ability to rapidly adapt to different environments.

Weed control is one of the most important components of cropping systems that results in significant yield and financial loss to growers if not properly performed. This scenario is exacerbated by the evolution of herbicide resistant weed populations, with 514 unique cases reported globally (Heap, 2020). Because of the overreliance on herbicides as the main weed management tool, resistance to multiple herbicide families within a single weed population is often documented (Neve et al., 2004). Multiple resistance represents a challenge to broad crop acreage production systems that depend on chemical weed management because of the lack of new herbicide molecules being marketed and the additional costs associated with non-chemical control methods.

Herbicide resistance mechanisms in weeds are typically classified in two categories: (a) modifications in the herbicide target enzyme (target-site resistance; TSR) and (b) mechanisms not involving the target enzyme (non-target-site resistance; NTSR). TSR is typically conferred by single major-effect alleles, whereas NTSR are believed to be conferred by multiple small-effect alleles (Jasieniuk et al., 1996; Délye, 2013), although this is not necessarily always the case (Yu et al., 2009b).

Physiological and biochemical alterations have been observed in weeds with NTSR, such as reduced herbicide absorption and translocation (Koger and Reddy, 2005), enhanced herbicide metabolism (Hall et al., 1997), and herbicide sequestration to the vacuole (Ge et al., 2010). However, the underlying physiological, biochemical, and genetic alterations conferring herbicide resistance is poorly understood.

Herbicide resistance in *Lolium* spp. populations has been widely documented. There are at least 125 reports of herbicide resistance in this genus to date, where multiple- and cross-resistance represent approximately 40% of the reports (Heap, 2020). In some regions of the world where environmental conditions for *Lolium* spp. development are ideal and there is an overreliance on herbicides as the main weed management tool, proportion of populations with multiple- and cross-resistance may be as high as 61% (Bobadilla, 2019). Herbicide resistance in *Lolium* spp. has been reported to 14 mechanisms of action, with an example of one population of *L. rigidum* from Australia with evolved resistance to seven mechanisms of action (HRAC/WSSA numbers 1, 2, 3, 8, 15, 13, and 23) (Burnet et al., 1994). *Lolium* spp. populations have evolved a variety of resistance mechanisms, including enhanced herbicide metabolism, reduced herbicide absorption and translocation, and protection-based resistance. Therefore, comprehensive reviews on the mechanisms of NTSR in *Lolium* spp. are needed.

In this article, we first provide an overview of NTSR mechanisms in weeds, with focus on grass species. We then review seminal and recent studies on NTSR in *Lolium* spp. It was not our goal to detail every single case of suggested NTSR in *Lolium* spp. Rather, we focused our efforts to compile the most relevant studies on NTSR in *Lolium* spp., what is known about the resistance mechanisms, and provide suggestions on how we can further our understanding of NTSR.

## NTSR Mechanisms in Weeds

### Reduced Herbicide Absorption

**FIGURE 1:**
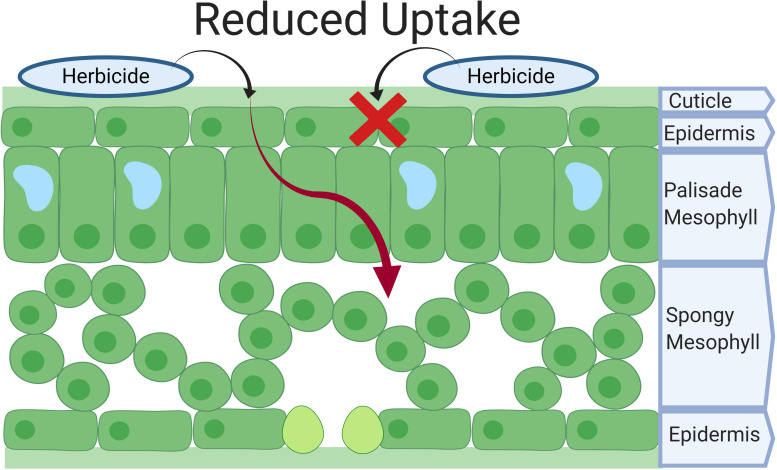
Herbicide absorption in *Lolium* spp. Herbicide molecules must overcome the cuticle and epidermis to reach the apoplast before cellular uptake. X represents a halt in the herbicide absorption and the red arrow represents the pathway to the mesophyll that the herbicide molecules would normally have without a reduction in absorption.

Upon herbicide application, herbicide droplets must land on the leaf surfaces and overcome a number of barriers before cellular uptake. This passive process largely depends on leaf surface characteristics, herbicide chemical properties, and their interactions. Is this review, we distinguish herbicide absorption from cellular uptake, where the former is the process of overcoming the physical barrier of leaves (i.e., cuticle) before the herbicide reaches the apoplast, and the latter is the movement of herbicide from the apoplast into plant cells. Herbicide resistant populations may exhibit reduced herbicide absorption, which is characterized by a reduction in the penetration through the cuticle before reaching the epidermis (Figure 1), whereas cell walls do not pose a considerable resistance to cellular uptake (Sterling, 1994). Reduced absorption is not a common NTSR mechanism, but has been documented in both eudicots and monocots to the herbicide groups synthetic auxins and 5-enolpyruvylshikimate-3-phosphate synthase (EPSPS) inhibitors, resulting in low resistance levels (Kohler et al., 2004; De Carvalho et al., 2012).

### Reduced Translocation and Vacuolar Sequestration

**FIGURE 2:**
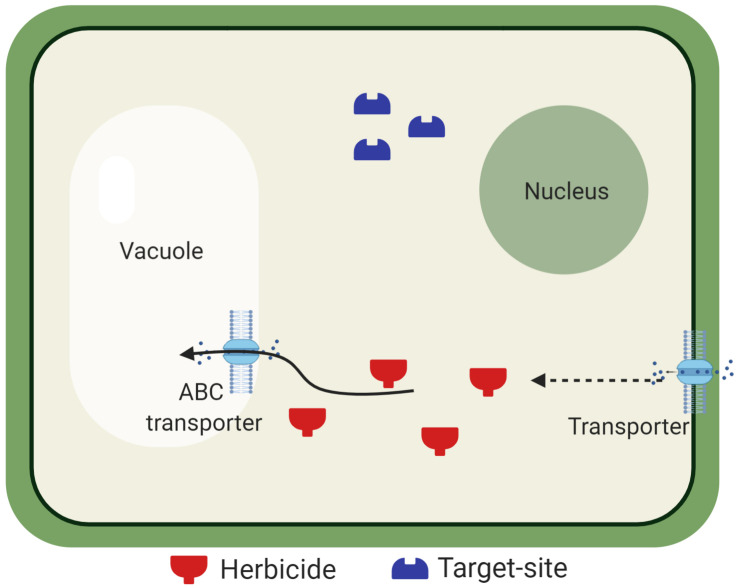
Reduced herbicide translocation due to vacuolar sequestration. After herbicide cellular uptake, herbicide molecules must reach the target site. Tonoplast-bound transporters are believed to be involved in the vacuolar sequestration of herbicides in resistant *Lolium* spp. populations, preventing the herbicide from inhibiting its target enzyme. Transporters are also located in the plasma membrane for apoplast sequestration.

Most herbicides must translocate from their absorption site in order to control weeds. Therefore, alterations of translocation patterns can diminish herbicide efficacy (Figure 2). Herbicide resistance due to reduced translocation has been documented in grass weed species, such as *Lolium* spp. and *Chloris elata* (Wakelin et al., 2004; Yu et al., 2007, 2009a; Bostamam et al., 2012; González-Torralva et al., 2012; Brunharo et al., 2016). The underlying genetic and physiological basis of this NTSR mechanism remains poorly understood (Yuan et al., 2007; Ge et al., 2010, 2014).

Plant vacuoles are organelles that have central roles in the cell homeostasis, are involved in osmotic adjustment, are reservoirs for ions and metabolites, and storage of xenobiotics (Marty, 1999). Studies have shown that transporters such as ATP-binding cassettes (ABC) are possibly involved in herbicide movement into the vacuoles (Nol et al., 2012; Ge et al., 2014; Tani et al., 2015). Because many herbicides must reach a target site localized within specific organelles, the vacuolar sequestration may prevent the herbicides from reaching the target site, as well as symplastic movement of the herbicide molecules.

Reduced herbicide translocation as a NTSR mechanism varies with environmental conditions, in particular temperature. Studies have shown that low temperature regimes can reduce the resistance levels by affecting the kinetics of vacuole sequestration (Devine et al., 1983; Vila-Aiub et al., 2005; Shaner, 2009). A paraquat-resistant *L*. *multiflorum* biotype from California, for instance, exhibited a GR_50_ (herbicide dose required to reduce plant biomass by 50%) 21 times greater when grown at 30/24°C than at 16/10°C. This population also exhibited enhanced protection against reactive oxygen species (ROS) (Brunharo and Hanson, 2019).

### Herbicide Metabolism

Herbicide metabolism refers to the degradation of herbicide molecules by endogenous plant enzymes. In some instances, this type of NTSR is non-specific, when a single enzyme may inactivate one or more herbicide within the same or different chemical classes (Iwakami et al., 2014b; Yu and Powles, 2014). Many aspects of the herbicide detoxification process are still unknown; however, key enzymes have been identified. Metabolism-based herbicide resistance occurs due to the increased activity of enzymes such as cytochrome P450’s (Vila-Aiub et al., 2005; Yun et al., 2005; Busi et al., 2011; Iwakami et al., 2014a), glutathione S-transferases (GST’s) (Reade et al., 2004; Cummins et al., 2011; Chronopoulou et al., 2017; Dücker et al., 2019), ABC transporters (Rea et al., 1998; Yuan et al., 2007; Tani et al., 2015), and glucosyltransferases (GT) (Cotterman and Saari, 1992; Yuan et al., 2007; Powles and Yu, 2010). The genetic mechanisms of the altered enzyme activity is not fully understood. Several hypothesis, however, may be inferred: (i) genetic modifications within the genes that encode metabolizing enzymes are involved, enhancing their activity; (ii) genetic modifications outside of the genes (e.g., in the promoter region or intragenic regions) enhance gene expression and, consequently, number of enzymes available to degrade herbicides; (iii) epigenetic changes occurred due to previous stressors (e.g., low rates of herbicides) that altered the epigenome, enhancing the expression of genes that encode metabolizing enzymes; (iv) and post-translational modifications of proteins enhance enzyme activity.

**FIGURE 3:**
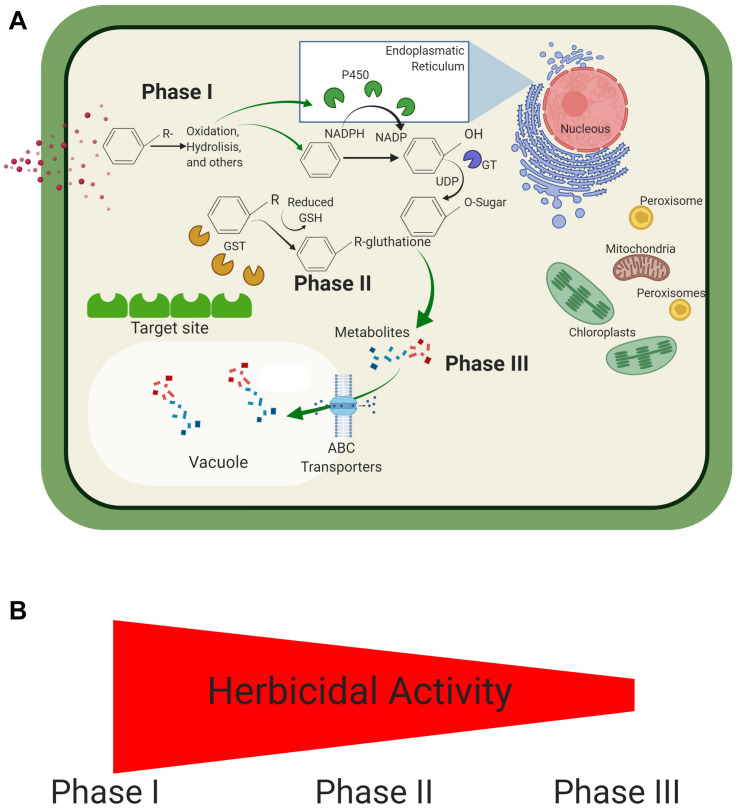
Herbicide metabolism in *Lolium* spp. An herbicide may be metabolized in three distinct processes, which typically occur in consecutive order. **(A)** Initially, the herbicide is subjected to a redox reaction to increase its hydrophilicity (Phase I). This metabolized herbicide may now be subjected to further processing in Phase II (e.g., conjugation). Metabolism may be concluded with the storage of metabolized compounds (Phase III). **(B)** The herbicidal activity decreases with the consecutive processing of herbicides.

Herbicide metabolism can be divided into three phases (Figure 3). The process starts after herbicide cellular uptake. Hydrophobic herbicide molecules are oxidized to a more hydrophilic metabolite, generally by P450’s (e.g., hydrolysis, oxidation, etc.; Phase I). Once the herbicide molecule is more hydrophilic, a conjugation reaction of the herbicide molecule may take place, and the herbicidal activity and hydrophobicity are further reduced (Phase II). Herbicides that already possess hydrophilic properties may be directly subjected to Phase II. Lastly, transport enzymes may recognize conjugated herbicide molecules before storage into vacuoles and cell walls (Phase III) (Yuan et al., 2007; Délye, 2013; Yu and Powles, 2014; Jugulam and Shyam, 2019). Some researchers also recognize a Phase IV of the herbicide metabolism process, where stored molecules are later utilized for plant metabolism (Rosinger et al., 2012).

Cytochrome P450’s are oxidoreductase enzymes that catalyze the hydroxylation, oxidation, and reduction, among other reactions, of substances in many organisms and are known to play a significant role in protecting plants from abiotic and biotic stresses (Mizutani and Sato, 2011). Plants have over 40 different families of cytochrome P450’s and are divided into four categories according to their primary function. P450 gene sequences occupy approximately 1% of the plant genome, reflecting their importance in plant defense and other functions such as synthesis and catabolism of plant hormones (Nelson and Werck-Reichhart, 2011; Mizutani, 2012; Pandian et al., 2020).

According to Guengerich (2018) and Pandian et al. (2020), P450 herbicide detoxification is known to happen in five steps: the first step consists in the herbicide binding to the heme group. In the second step, the substrate binding induces the electron transfer from NADPH by P450 reductase; the third step consists in oxygen binding to the ferrous cytochrome forming a complex; in the fourth step, the P450 reductase will release another set of electrons to the ferrous cytochrome-dioxygen complex forming a short lived “peroxo” complex that is rapidly protonated forming a water and an iron–oxo complex; the last step consists on the complex binding to the organic herbicide molecules forming an oxidized product.

Many studies that reported enhanced herbicide metabolism as the resistance mechanism did so by indirect means. Typically, a P450 inhibitor is applied either before or with the herbicide being tested, with the expectation that the P450 inhibitors would reverse the resistance phenotype. For instance, Christopher et al. (1994) pre-treated chlorsulfuron-resistant *L. rigidum* with malathion (a P450 inhibitor) and observed that the resistant population responded similarly to the susceptible. More recently, cytochrome P450s were identified to confer NTSR resistance in *Lolium* spp. populations from Argentina after the authors pre-treated plants with malathion, 1-aminobenzotriazole, and piperonyl butoxide. P450 inhibitors are a widely used approach to identify the role of P450s in herbicide resistance (Busi et al., 2017; Zhang et al., 2017; Yanniccari et al., 2020).

The enzyme super-family of GSTs is also involved in herbicide detoxification in plants. In maize, for instance, GST’s represent more than 1% of soluble proteins in leaves (Edwards et al., 2000). GST’s catalyze the conjugation of many hydrophobic and electrophilic substrates with the tripeptide glutathione (Edwards et al., 2000). GST’s are likely to be involved in the compartmentalization of herbicides by conjugating glutathione with herbicide molecules and facilitating the recognition of glutathione transporters making them potential participants in reduced translocation-based resistance (Reade et al., 2004).

The ABC superfamily is another large group of proteins that is responsible to mediate a wide range of transport functions in plants (Theodoulou, 2000). ABC transporters can play a role in the transport and movement of many compounds such as peptides, sugars, lipids, heavy metal chelates, polysaccharides, alkaloids, steroids, inorganic acids, and glutathione conjugates; these transporters can be highly specific and able to transport a large variety of compounds (Higgins, 1992). Research has shown that ABC transporters may actively transport and compartmentalize herbicide conjugates and metabolites (Powles and Yu, 2010; Gaines et al., 2020). ABC transporters have been hypothesized to be involved in the glyphosate resistance mechanism in *Lolium* spp. (Ge et al., 2012).

Glycosylation mediated by GT’s may alleviate stresses caused by xenobiotics in plants (Bowles et al., 2005). In grasses, GT’s are known to be responsible for their tolerance to synthetic auxins via glycosylation (Devine et al., 1993). In many *Lolium* spp. studies, genes that encode GT’s have been identified as potential players in the resistance mechanisms to several herbicides (Gaines et al., 2014; Busi et al., 2018; Dücker et al., 2019).

### Protection-Based Resistance

Protection-based herbicide resistance is conferred by endogenous enzymes that counteract the damaging effect of reactive molecules that were elicited by the action of an herbicide. The most widely studied enzymes are those of the Halliwell-Asada cycle, which are involved in the protection of plant cells against oxidative damage (Délye, 2013), and include superoxide dismutase, ascorbate peroxidase, and glutathione reductase. Many herbicides elicit the overproduction of ROS which can induce oxidation of proteins, DNA, and lipids, resulting in cellular damage and causing cellular leakage. A plant that can avoid or reduce the presence of ROS can minimize the stress caused by herbicides. An *Alopecurus myosuroides* population with multiple resistance to photosystem II (PSII) and acetyl CoA carboxylase (ACCase) inhibitors exhibited an enhanced activity of enzymes involved in the cellular protection against toxic organic hydroperoxides (Cummins et al., 1999). However, there are few documented cases of protection-based resistance, and detailed information on its role as a secondary mechanisms of resistance is limited. If individuals in a population exhibit enhanced protection against ROS, then it would be expected that reduction in efficacy of many herbicide classes would be observed.

## Herbicide Resistance in *Lolium* spp. and Their Mechanisms of NTSR

### Resistance to ACCase Inhibitors

**FIGURE 4:**
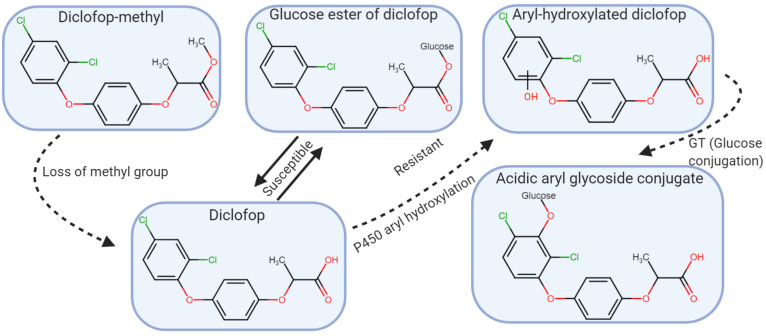
Diclofop-methyl metabolism in susceptible and resistant plants. Diclofop-methyl is demethylated, and converted to the active form of the herbicide. In resistant plants, diclofop undergoes an aryl hydroxylation reaction likely mediated by P450, followed by a conjugation reaction to acidic aryl glycoside of diclofop. In susceptible plants, diclofop is reversibly converted to a glucose ester conjugate (modified from Powles and Holtum, 1994).

Herbicides in the aryloxyphenoxy-propionate (FOP’s), cyclohexanedione (DIM’s), and phenylpyrazoline (DEN) chemical families (HRAC/WSSA Group 1) inhibit ACCase, an enzyme in the biosynthetic pathway that produces fatty acids, which are required for lipid production needed for cell membranes (Hoppe, 1989). The binding site is a 400-amino acid fragment of the carboxyltransferase (CT) domain in ACCase (Nikolskaya et al., 1999; Takano et al., 2021). Herbicides in these families are extremely effective for grass control and in general, the chloroplastic ACCase from broadleaf plants is not sensitive to ACCase inhibiting herbicides (Konishi and Sasaki, 1994). In tolerant grasses, the herbicides are metabolized to non-toxic products or have insensitive ACCase (Shimabukuro, 1985; Duke and Kenyon, 1988; Zimmerlin and Durst, 1992). Some of the herbicides are selective and can be used in cereal crops while others are non-selective. For example, wheat (*Triticum aestivum*) is tolerant to diclofop-methyl and clodinafop-propargyl but not to fluazifop-p-butyl, quizalofop-p-ethyl, clethodim, and sethoxydim (Shaner, 2014). In susceptible plants and in wheat, diclofop-methyl is bioactivated by hydrolysis to form the phytotoxic diclofop acid (Figure 4). In wheat, the acid is detoxified by aryl hydroxylation catalyzed by a P450 monooxygenase followed by glucosylation to produce a non-toxic glucose conjugate (Shimabukuro, 1985).

Resistance to ACCase inhibitors in *Lolium* spp. is common with reports from all continents except Antarctica. Diclofop resistant *L. rigidum* was reported in Australia in 1982 (Heap and Knight, 1982) and *L. multiflorum* in Oregon in 1987 (Stanger and Appleby, 1989). Subsequently, resistance has been reported in *Lolium* spp. to all herbicides in Group 1. Although, resistance to clethodim is less common. Resistance to one of the herbicides in this group does not necessarily lead to cross-resistance with other members of the group. Target site resistance is due to a single point mutation in the ACCase gene with at least five different mutations reported with some mutations providing resistance to all three families (Powles and Yu, 2010; Takano et al., 2021).

Non-target-site resistance to diclofop in *L. rigidum* was reported in Australia in 1991 (Holtum et al., 1991). The researchers did not believe that the 10% difference in metabolism between resistant and susceptible plants was enough to produce a 30-fold difference in sensitivity at the whole plant level. The authors suggested that metabolism plus membrane repolarization might be responsible for resistance. Other researchers also proposed that membrane depolarization results from the application of ACCase inhibitors and that resistant plants were able to recover from this effect (Devine and Shimabukuro, 1994; Shimabukuro and Hoffer, 1997). However, the membrane depolarization observed in plants treated with ACCase inhibitors may be considered a secondary effect, as was determined the target is the CT-domain of ACCase (Nikolskaya et al., 1999). Further research on resistant *Lolium* spp. populations showed that enhanced metabolism via P450 followed by conjugation by GST enzymes were responsible for resistance (Preston et al., 1996; Preston and Powles, 1998; Cocker et al., 2001; De Prado et al., 2005). De Prado et al. (2005) also reported reduced absorption of diclofop and greater epicuticular wax density in one resistant biotype of *L. rigidum*.

### Resistance to AHAS Inhibitors

There are five herbicide families (HRAC/WSSA Group 2) that inhibit acetohydroxyacid synthase (AHAS), also referred to as acetolactate synthase (ALS), the first enzyme in the biosynthetic pathway for the production of the branched chain amino acids, isoleucine, leucine, and valine. The families are imidazolinones, pyrimidinyl-thiobenzoates, sulfonylamino-carbonyl-triazolinone, sulfonylureas, and triazolo-pyrimidines. The herbicides are used in nearly all cropping systems with major differences in their selectivity, spectrum of control, and residual activity.

Similar to the ACCase inhibitor herbicides, resistance to AHAS inhibitors in *Lolium* spp. has been reported on every continent except Antarctica. Most of the resistant populations were identified in cereal cropping systems with some identified in other crops or in non-crop areas such as roadsides. Initially, TSR was reported to be the most common resistance mechanism with multiple different point mutations responsible for resistance (Tranel and Wright, 2002). However, there are many cases of NTSR AHAS resistance in *Lolium* spp. reported to be due to enhanced metabolism. Further, TSR or NTSR to one AHAS inhibiting herbicide does not necessarily endow resistance to another herbicide even within the same chemical family.

**FIGURE 5:**
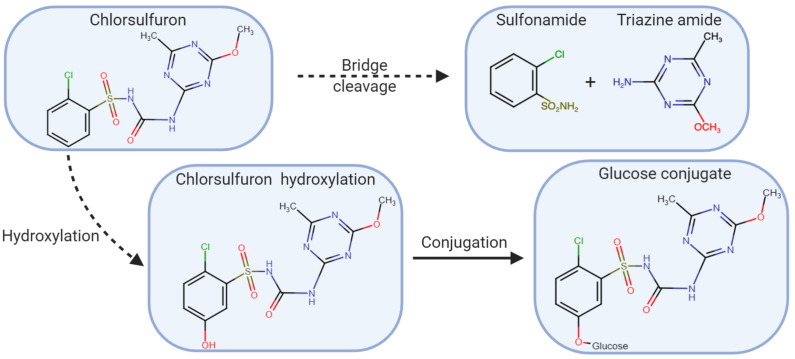
Chlorsulfuron metabolism in *Lolium rigidum*. The herbicide may be hydroxylated followed by conjugation with glucose, or cleaved, producing sulfonamide and triazine amide (adapted Cotterman and Saari, 1992).

In studies conducted on *L*. *rigidum*, metabolism of chlorsulfuron, a sulfonylurea herbicide, occurred more quickly in the resistant biotype compared to the susceptible biotype (Christopher et al., 1991, 1992). Using high-pressure liquid chromatography (HPLC), the major metabolite co-eluted was the glucose-conjugate metabolite previously identified in chlorsulfuron tolerant wheat (Figure 5; Christopher et al., 1991). In another study using a different chlorsulfuron resistant *L. rigidum* biotype, the major metabolite identified was the glucose conjugate of hydroxyl-chlorsulfuron (Cotterman and Saari, 1992). In the resistant biotype, 50% of the chlorsulfuron was metabolized within 2 h compared to 10% in the susceptible biotype. The percentage of the glucose conjugate occurred more rapidly and to a greater level in the resistant biotype compared to the susceptible biotype. The researchers further showed that chlorsulfuron metabolites were not AHAS inhibitors so the differences in rate and level of chlorsulfuron metabolism were responsible for resistance. In many other studies, resistance due to enhanced metabolism resistance was based on indirect evidence. In these studies, a cytochrome P450 inhibitor, such as malathion (Preston et al., 1996; Yu et al., 2009a) or chlorpyrifos (Liu M. et al., 2016), was applied. In these studies, resistance was overcame with the addition of the P450 inhibitor, implicating herbicide metabolism as the mechanism of resistance.

### Resistance to Microtubule Assembly Inhibitors

Herbicides that inhibit the assembly of microtubules do so by binding to α or β-tubulin (HRAC/WSSA Group 3) leading to loss of microtubule structure and function in the process of mitosis. Microtubules are required for the spindle apparatus, which separates sister chromatids during mitosis (Molin and Khan, 1997). This loss of function prevents cell division and cell wall formation. The most widely used herbicides with this mechanism of action are in the dinitroaniline chemical family, which includes the herbicide trifluralin.

Trifluralin resistant *L. rigidum* populations have been reported in Australia. In most cases, the resistant populations were found in cereal cropping systems. Several of the populations were reported to be resistant to other herbicides. In some cases, the mechanism of resistance was due to single point mutation in the α-tubulin gene, where four unique point mutations have been identified that provide resistance to trifluralin (Chen et al., 2018; Chu et al., 2018; Fleet et al., 2018).

The only reports of trifluralin NTSR are from studies conducted in populations collected in Western Australia, which confirmed NTSR via enhanced metabolism (Chen et al., 2018). Using thin-layer chromatography (TLC) and HPLC analyses, more trifluralin was metabolized in a resistant population compared to a susceptible population. Because metabolites were not identified, the specific degradation pathway was not determined. However, in previous studies with one of the resistant populations, resistance was reversed when a P450 inhibitor was applied indicating that a P450 enzyme might be involved with the enhanced metabolism (Busi et al., 2017). In addition, in this study, some plants evolved both TSR and NTSR to trifluralin.

### Resistance to Photosystem II Inhibitors

Several different herbicide chemical classes including triazines, triazinones, and ureas (HRAC/WSSA Groups 5 and 6) inhibit Photosystem II (PSII). The PSII complex is located within the thylakoid membranes of chloroplasts and contains two proteins, D2 and D1 (Fuerst and Norman, 1991). Once a PSII inhibiting herbicide binds, it blocks the transfer of electrons from plastoquinone Q_A_ in D2 to plastoquinone Q_B_ in D1, which prevents CO_2_ fixation and production of ATP and NADPH. Blocking electron transport leads to production of ROS, which destroy cell integrity.

The first documented case of herbicide resistance was in the dicotyledonous species common groundsel (*Senecio vulgaris* L.) to the PSII inhibitor simazine, a triazine herbicide (Ryan, 1970). Most often, the mechanism of resistance to PSII inhibitors is reported to be a mutation in the D1 protein in the PSII complex. However, there are some cases where resistance is NTS. In these cases, enhanced metabolism is reported to be responsible for resistance. Photosystem II inhibitor resistant *L. rigidum* populations have been reported in crop and non-crop sites in Australia, Israel, and Spain and *L*. *multiflorum* populations in United Kingdom cereal crops (Heap, 2020).

Metribuzin (HRAC/WSSA Group 5) is in the triazinone chemical family of PSII inhibitors. Metribuzin controls both grass and broadleaf weeds. In some tolerant species such as wheat, metribuzin is detoxified to polar N-glucoside metabolites (Devlin et al., 1987). Metribuzin resistance in a multiple-resistant *L*. *rigidum* population in Australia was due to enhanced metabolism (Ma et al., 2020). In a time course study, unidentified polar metabolites of metribuzin were found in both susceptible and resistant plants at each time point but were greater in the resistant plans (Busi et al., 2017). Based on the results of a dose response study with the addition of a P450 inhibitor, which reversed resistance, the authors suggested that the mechanism of resistance likely involved cytochrome P450 monooxygenases.

Chlorotoluron (HRAC/WSSA Group 5) is in the substituted urea chemical family of PSII inhibiting herbicides but has a different binding behavior compared to other herbicide classes in Group 5 (Shaner, 2014). Chlorotoluron is used to control grass and broadleaf weeds in cereals. In tolerant plants, the major degradation is through N-dealkylation and oxidation of the ring-methyl group with conjugation to glucose (Gonneau et al., 1988).

Similar studies on the mechanism of chlorotoluron resistance in *L. rigidum* populations were conducted in Australia (Burnet et al., 1993; Preston and Powles, 1996) and Spain (De Prado et al., 1997). Based on HPLC analysis, Burnet et al. (1993) found that resistant plants metabolized chlorotoluron more quickly than the susceptible plants. De Prado et al. (1997) used TLC and reported greater metabolism at 48 hr in the resistant versus susceptible plants. Both groups conducted studies using monooxygenase inhibitors to overcome resistance, which supported the premise that cytochrome P450 enzymes could be involved in degradation. In a follow up study on one of the resistant Australian populations, chlorotoluron was metabolized via two paths, which resulted in either ring-methyl hydroxylation or N-demethylation (Preston and Powles, 1997). In light, metabolism via ring-methyl hydroxylation increased significantly while N-demethylation did not. The products of ring-methyl hydroxylation were conjugated to glucose. The results indicated that the ring-hydroxylation was the major detoxification pathway and that N-demethylation was less important. The metabolism via both pathways was greater in chlorotoluron resistant plants than in susceptible plants. The authors suggest that two different enzymes are involved with enhanced metabolism of the resistant biotype because of the differences in the induction of the two pathways in response to light.

### Resistance to Glyphosate

Glyphosate [*N*-(phosphonomethyl)glycine, HRAC/WSSA Group 9] is the most widely used herbicide in the world in agricultural and non-agricultural areas. It inhibits EPSPS, preventing biosynthesis of aromatic amino acids for plant metabolism (Shaner, 2014). Glyphosate uptake by plant cells may be active or passive, with the active uptake being facilitated by membrane-bound phosphate transporters (Hetherington et al., 1998). Several properties make glyphosate a unique and important tool: it is a non-selective, systemic, slow-acting, post-emergence, and relatively non-expensive herbicide (Duke et al., 2018). Glyphosate is hydrophilic (log K_ow_ at pH 7 = of −3.1), a weak acid, and exhibits slow metabolic degradation in most plants or not at all, which makes it possible for glyphosate to be transported through the phloem and the xylem and move to meristems where amino acid synthesis is most required (Duke et al., 2018). In 1996, *L. rigidum* was the first species to have a confirmed glyphosate resistant population (Pratley et al., 1996).

Most cases of NTSR to glyphosate in *Lolium* spp. are due to reduced translocation, with more than 20 reports to date in several countries, including Australia, Brazil, Chile, France, Italy, Japan, New Zealand, Portugal, Spain, and the United States (Mississippi and Oregon) (Ferreira et al., 2006; Michitte et al., 2007; Perez-Jones et al., 2007; Yu et al., 2007; Nandula et al., 2008; Ge et al., 2012; Ghanizadeh et al., 2015b; Fernández-Moreno et al., 2017; Kurata et al., 2018). Some authors also reported lower spray retention and foliar uptake from the abaxial leaf surface, along with reduced translocation (Michitte et al., 2007).

Reduced glyphosate translocation in resistant plants keeps the herbicide in the source leaves, away from the meristematic tissue, enabling survival after treatment (Kurata et al., 2018). Susceptible biotypes commonly translocate glyphosate out of the treated leaves into non-treated leaves, meristematic tissues, stems and roots (Lorraine-Colwill et al., 2002; Wakelin et al., 2004; Perez-Jones et al., 2007; Yu et al., 2009a). Different populations exhibit a wide range of resistance levels, ranging from three- to 25-fold compared to susceptible populations (Ghanizadeh et al., 2015b; Kurata et al., 2018).

Reduced glyphosate movement in glyphosate resistant plants may occur *via* four mechanisms: (i) modification in a putative phosphate transporter located in the plasma membrane, (ii) an active transporter pumps glyphosate into the vacuole, (iii) glyphosate pumped out of the cell into the apoplast through an active transporter, (iv) glyphosate pumped out of the chloroplast by a transporter in the chloroplast envelope (Shaner, 2009). However, to date, these mechanisms remain hypothetical, and no transporter has been identified to confer glyphosate resistance in weeds.

A modification in a phosphate carrier protein has been proposed as a resistance mechanism to glyphosate (Shaner, 2009; Roso and Vidal, 2010). It has been shown that glyphosate does not readily move across a laboratory made semi-permeable membrane (Takano et al., 2019) and cellular uptake may be inhibited in the presence of phosphate (Hetherington et al., 1998). These results provide evidence that glyphosate is taken up by the cell through a phosphate transporter. Therefore, a putative modification in such a transporter would keep glyphosate out of the cell. However, a possible modification in the carrier has not been found to date in *Lolium* spp.

The second possible mechanism, a transporter pumping glyphosate into the vacuole has been the hypothesis with the most evidence found to date. In a study using ^31^P nuclear magnetic resonance, vacuolar sequestration of glyphosate in populations of *Lolium* spp. from four different countries was strongly correlated with reduced translocation, and thus, reduced entry of glyphosate into the phloem (Ge et al., 2012). The authors concluded that glyphosate sequestration into the vacuole appeared to be unidirectional, meaning that once inside the vacuole, efflux through the tonoplast does not seem to be significant. The authors hypothesized that glyphosate is transported into the vacuole through an unidentified tonoplast-bound ABC transporter (Ge et al., 2012; Sammons and Gaines, 2014). To date, only a few studies have investigated the vacuolar sequestration and its association with reduced translocation of glyphosate. However, a few candidate genes have been identified. Glyphosate movement across the tonoplast is reduced under low temperatures (Ge et al., 2011). Studies in *Lolium* spp. have used low temperature treatments after glyphosate application as indirect evidence that glyphosate was sequestered into the vacuole (Vila-Aiub et al., 2013; Ghanizadeh et al., 2015a). *Lolium* spp. populations evaluated in other studies had reduced herbicide translocation as the mechanism of resistance when grown at ambient temperatures (Lorraine-Colwill et al., 2002) of 26/12°C (Ghanizadeh et al., 2015b). When grown at 9°C after glyphosate application, the resistant population responses were similar to the susceptible population. In comparison, a glyphosate resistant *L. multiflorum* with an *EPSPS* Pro_106_Ser amino acid substitution was not made sensitive to glyphosate with cold acclimation (Collavo and Sattin, 2012; Sammons and Gaines, 2014). However, since low temperature is also the same method used to identify possible metabolism based resistance, more research would need to be done to rule out this hypothesis and elucidate the effects of temperature on the vacuolar sequestration of glyphosate.

Although most studies of resistant populations with reduced translocation did not further investigate the underlying genetic basis of the NTSR, it is very likely that they also had vacuolar sequestration, as enhanced glyphosate metabolism has rarely been identified to date (however, see Pan et al., 2019; McElroy and Hall, 2020).

Reduced glyphosate translocation generally results in higher resistance levels than alterations in the EPSPS enzyme (Preston and Wakelin, 2008; Bostamam et al., 2012). It has been suggested that two or more mechanisms of resistance in the same population, can result in a higher level of resistance (Ghanizadeh et al., 2015b). As *Lolium* spp. are obligate outcrossing species, different mechanisms of resistance and resistance to different herbicides may accumulate due to cross-pollination (Yu et al., 2007).

No evidence of glyphosate being pumped out of the cell into the apoplast, nor being pumped out of the chloroplast envelope has been found to date. Glyphosate transport through membranes has been observed as being unidirectional by importers (Ge et al., 2013). Once glyphosate enters the chloroplast, it has been assumed that it cannot return to the cytoplasm (Sammons and Gaines, 2014). An upregulated gene was found to be related to ABC transporter A family member 7 (*ABCA7*) in a NTSR glyphosate resistant *L. multiflorum* population (Cechin et al., 2020), which its subcellular location is in the plasma membrane in *Arabidopsis thaliana* (Benschop et al., 2007). Further validation studies could help determine if the identified transporter gene is responsible for glyphosate resistance.

Enhanced glyphosate metabolism has not been found to be a resistance mechanism in *Lolium* spp.; however, Fernández-Moreno et al. (2017) found that susceptible and resistant populations of *L. perenne* and *L. multiflorum* metabolized glyphosate to aminomethylphosphonic acid (AMPA) and glyoxylate. The authors concluded that the final concentrations of the metabolites were small and unlikely to be biologically meaningful. AMPA is a very weakly phytotoxic compound (Gaines et al., 2020) and glyoxylate is a non-toxic compound (Rueppel et al., 1977), therefore rapid degradation to those substances should provide glyphosate resistance. In a RNA-seq study comparing a susceptible and a NTSR population, the candidate gene list included genes related to glycosyltransferases (Cechin et al., 2020). Glycosyltransferases are important for crop tolerance; however, their role in herbicide resistance in weeds is still not well understood and glucosylation of glyphosate as a mechanism of NTSR has yet to be identified (Rigon et al., 2020). Future studies with reverse genetics to evaluate candidate genes are required.

### Resistance to Glufosinate

Glufosinate (HRAC/WSSA Group 10), the only member of this herbicide group, controls weeds by inhibiting the glutamine synthetases, key enzymes in the nitrogen assimilation in plants. Inhibition of glutamine synthetase reduces the amount of amino donors for the glycolate pathway, breaking the transamination reaction of glyoxylate to glycine in the photorespiratory cycle (Wild and Wendler, 1993). This imbalance leads to accumulation of glyoxylate, which is a strong inhibitor of the ribulose-1,5-bisphosphate carboxylase activase, necessary for the proper functioning of ribulose-1,5 bisphosphate carboxylase/oxygenase. Consequently, photosynthesis is inhibited (Wendler et al., 1992; Wild and Wendler, 1993; González-Moro et al., 1997), causing accumulation of ROS and cell death (reviewed by Hess, 2000, and more recently by Takano and Dayan, 2020).

There are, overall, a limited number of glufosinate resistant weed populations, likely associated with the limited use of this herbicide until recent years. More recently, however, particularly because of patent expirations and increased adoption of glufosinate resistant crops, the number of resistant populations has increased and this trend is likely to continue. Glufosinate resistance in *L. multiflorum* was first identified in 2009 in hazelnut (*Corylus avellana*) orchards in Oregon, where resistant populations exhibited up to 2.7-fold reduced response to glufosinate compared to a known susceptible population (Avila-Garcia and Mallory-Smith, 2011; Avila-Garcia et al., 2012). Later, research by Brunharo et al. (2019) indicated that there are multiple mechanisms of glufosinate resistance in the Oregon populations. The authors studied two resistant populations, one of them exhibited enhanced glufosinate metabolism, and the other did not. No differences in absorption, translocation of glufosinate, or differential gene expression of three GS isoforms studied were observed. The metabolites produced by glufosinate resistant *L*. *multiflorum* were not identified. Several plant species have been identified that may metabolize glufosinate, including tobacco and carrot (Dröge et al., 1992), producing several stable and unstable compounds with reduced herbicidal activity (Droge-Laser et al., 1994). Current research is underway to identify the genetic basis of glufosinate resistance in *L. multiflorum*.

### Resistance to Very-Long Chain Fatty Acid Inhibitors

Very-long chain fatty acid (HRAC/WSSA Group 15) inhibitors (e.g., flufenacet, metolachlor, and pyroxasulfone) prevent biosynthesis of very-long chain fatty acid although a specific target enzyme or enzymes within the pathway have not been identified. Trenkamp et al. (2004) reported that flufenacet inhibits multiple elongases in the pathway.

Rapid metabolism of flufenacet via glutathione conjugation is found in tolerant crops with flufenacet-glutathione being the first major metabolite (Bieseler et al., 1997). Activity rates of GST were greater in maize, a tolerant crop, than in sensitive species, supporting the role of this enzyme in the breakdown of flufenacet in plants (Kreuz et al., 1989).

Resistance to flufenacet has been reported in *L. multiflorum* in France and United States (Gersdorf, 2009; Rauch et al., 2010; Liu M. et al., 2016; Bobadilla, 2019; Dücker et al., 2019). Most of the resistant populations were found in either cereal or grass seed cropping systems and were resistant to other herbicides (i.e., exhibited cross- and multiple-resistance). Liu M. et al. (2016) suggested that resistance in populations from Oregon was based on enhanced metabolism.

Pyroxasulfone resistance has been artificially created in *L*. *rigidum* populations under laboratory conditions after recurrent low-rate herbicide (Busi et al., 2012). These populations were subjected to three cycles of an increasing rate of pyroxasulfone, and the resistance phenotype has been attributed to an enhanced rate of herbicide metabolism (Busi et al., 2018). A field population of *L. rigidum* evolved pyroxasulfone resistance in Australia (Brunton et al., 2019).

**FIGURE 6:**
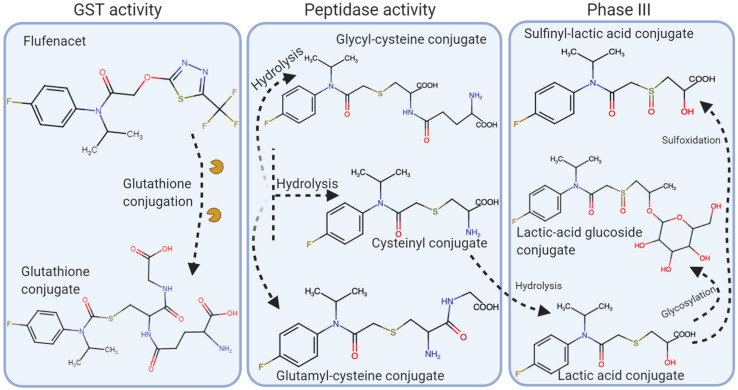
Flufenacet metabolism in *Lolium* spp. Herbicide conjugation is initially performed by GST’s. The conjugate molecule is hydrolyzed and processed by peptidases, which will eventually be further processed in the Phase III of the metabolism pathways (adapted from Dücker et al., 2019).

Studies conducted by Dücker et al. (2019) found that flufenacet resistance in *L. multiflorum* populations from France, the United Kingdom, and Washington State, United States, was due to enhanced metabolism. Flufenacet was degraded more quickly in resistant plants than in susceptible plants with some variation among the susceptible and resistant tested populations (Figure 6). In sensitive populations at 22°C, times for 50% degradation (D_50_) of flufenacet were 7 to 12 h whereas in the resistant populations the D_50__s_ were 0.09 to 0.41 h. At 12°C, the D_50__s_ were 18.5 to 46 h for the susceptible populations and 1.3 h for the resistant populations. A flufenacet-glutathione conjugate was found to be the first metabolite in the degradation pathway. GST activity was greater in the resistant plants than in susceptible populations. Two additional metabolites were identified in the resistant plants during the time course study. At 24 h, metabolites that were likely the result of secondary conjugation with malonyl or glycosyl were detected.

### Resistance to Photosystem I Electron Diverters

Paraquat and diquat are non-selective herbicides (WSSA/HRAC Group 22) that function as preferential electron acceptors in the Photosystem I (PSI), where electrons from ferredoxin are diverted from their regular path, producing ROS that cause lipid peroxidation and tissue necrosis (Summers, 1980). Throughout this section, the focus will be given on paraquat, as more in-depth studies on the NTSR mechanisms for this herbicide are available.

Paraquat cellular uptake is facilitated by plasma membrane-bound polyamine transporters (Hart et al., 1992), likely because of the similar chemical structure the herbicide shares with these natural substrates (Fujita and Shinozaki, 2014). Once the plasma membrane barrier is overcome, paraquat must reach its target site located in the chloroplast, more specifically in the thylakoid membrane. It is unclear whether paraquat transport through the chloroplast’s double-membrane, particularly the inner, less-permeable membrane, is passive or active. Results from Li et al. (2013) suggest that an L-type amino acid (LAT) transporter localized to the Golgi apparatus facilitates paraquat movement into the chloroplast. LAT transporters are involved in the intracellular movement of LAT, polyamines, and organocations in mammals (Jack et al., 2000), and the authors suggested that LAT transporters facilitate the movement of paraquat to the chloroplast.

Because paraquat does not have a target site enzyme associated with its mechanism of action, resistance to paraquat has always been associated with NTS. Resistance to paraquat has been proposed to be either because of vacuolar sequestration of the herbicide or enhanced protection against ROS, where the former typically confers higher resistance levels. Although there are many reports of differential response to PSI inhibitors in populations of *Lolium* spp. (Faulkner, 1974; Harvey et al., 1978), the first field-selected case of PSI resistance was not identified until 2002 (Yu et al., 2004).

*Lolium rigidum* was the first member of the *Lolium* spp. complex to exhibit PSI inhibitor resistance (Yu et al., 2004) from a vineyard in South Africa. The resistant population exhibited 30-fold reduced translocation compared to a known susceptible population. The authors suggested that the mechanism of paraquat resistance involved enhanced vacuolar sequestration of the herbicide, supported by the fact that resistance could be reversed by plant incubation under low temperatures, as is observed for paraquat resistance in other species (Purba et al., 1995). Later inheritance studies in other populations suggested that a major nuclear gene confers paraquat resistance, as the phenotype followed Mendelian segregation (Yu et al., 2009b).

Paraquat resistance in *L. multiflorum* was first observed in 2015, in a population from a prune orchard in California (Brunharo and Hanson, 2017). No differences were observed in paraquat metabolism or absorption when the resistant and susceptible biotypes were compared when grown at 30/24°C (Brunharo and Hanson, 2019). However, significant differences in paraquat translocation were detected, where the resistant biotype translocated less paraquat than the susceptible in a light-manipulated environment. After paraquat application, the resistant biotype exhibited a transient inhibition of photosynthesis, suggesting a mechanism of response to the herbicide once inside the plant cells. Furthermore, at low paraquat doses, there was no damage observed to thylakoid membranes of treated plants, suggesting a constitutive mechanism to cope with the herbicide, whereas at higher paraquat doses significant damage was observed. The authors concluded that paraquat resistance was due to vacuolar sequestration of the herbicide, because pre-treatment of leaf tissues with a tonoplast-bound polyamine transport inhibitors reversed the resistance. When this population was acclimated to low temperatures 16/10°C, paraquat resistance was no longer observed (Brunharo and Hanson, 2019). This population also exhibited an enhanced ability to detoxify ROS. To the best of our knowledge, there are no reports of PSI resistance in *L. perenne*. The physiological mechanisms involved in the paraquat resistance reversal under low temperatures have not been elucidated. However, one could hypothesize that, if the resistance mechanism depends on enzyme kinetics of transport proteins, then low temperatures will reduce the rate of enzyme reactions.

## Strategies to Uncover NTSR

Scientists have acquired a plethora of information on target-site resistance. The large amount of information on TSR may be attributed to the fact that, when herbicide resistance is believed to have evolved in a weed population, looking for changes in the target site can be successfully achieved relatively quickly in the laboratory today. Basic understanding of NTSR mechanisms, conversely, is still in its early stages of discovery, and limited advances on the genetic basis have been achieved to date (Yu and Powles, 2014; Wang et al., 2017; Oliveira et al., 2018; Van Etten et al., 2020). It is believed that recurrent selection by low herbicide dosages plays a major role in the stacking of multiple small-effect alleles conferring NTSR (Délye, 2013), and the interactions among the resistance alleles may play an important role in the resistance level (Renton et al., 2011).

Although less often acknowledged in the literature, stresses caused by sub-lethal herbicide doses may play an important role in the evolution of NTSR (as reviewed by Dyer, 2018) by inducing systemic stress responses that lead to genetic and epigenetic changes upon which selection can act (Ram and Hadany, 2014; Hu et al., 2016; Kim et al., 2017). These epigenetic modifications driven by environmental cues during the plant life cycle can be inherited and remain stable for as long as the stressors remain (Cubas et al., 1999; Hsieh et al., 2016).

Identifying the underlying genetic basis of NTSR is a challenging task that takes time and resources. To date, several examples of these attempts are available in the literature, and scientists have been able to identify candidate genes efficiently (see discussion below). Further validation of candidate alleles via functional analysis are rare; however, these are the ultimate approaches necessary to relate the genotype with the resistance phenotype.

High-throughput sequencing technologies, associated with the exponential cost reduction of these technologies, have enabled researchers to acquire massive amounts of data, not only for model species (e.g., *A. thaliana*) but also for non-model organisms, as is the case of *Lolium* spp. This enormous data quantity makes possible genome-wide interrogations of causal genetic features associated with traits of interest. Although such interrogations are common place in other disciplines, limited research has explored the underlying basis of NTSR in weed populations. Different methods have different benefits and drawbacks, and existing knowledge of the target organism will aid in the choice of the most appropriate approach to study NTSR.

Transcript expression quantification has been used in the field of weed science to investigate the mechanisms of NTSR. Prior information on the potential enzymes and herbicide metabolites involved in the NTSR are essential when low-throughput methods are adopted to study the resistance mechanisms (e.g., real-time quantitative polymerase chain reaction), as these approaches are very laborious and time consuming (Iwakami et al., 2014a,b; Guo et al., 2019). When limited information about the physiological and biochemical aspects of a resistance phenotype is available, high-throughput sequencing approaches (i.e., RNseq) may be a better option. Careful consideration of the experimental design plays an important role in the success of the RNA-seq analysis (Giacomini et al., 2018). Given the limited genomics resources currently available in most weed species, a *de novo* reference transcriptome assembly is the first step in a differential expression analysis (Gaines et al., 2014; Keith et al., 2017; Zhao et al., 2017). Another consideration when designing RNA-seq studies is the genetic background control of the experimental units, as it might determine the number of differentially expressed contigs identified (as reviewed by Giacomini et al., 2018). It is recommended that crosses be performed before final RNA extraction, so that researchers may take advantage of recombination and reduce the number of candidate genes. Following quantification of differentially expressed contigs, further analysis is necessary to identify candidate genes, and typically require a prior physiological and biochemical knowledge of the phenotype. By filtering contigs unlikely to be involved in pyroxsulam resistance in *Lolium* spp. (Duhoux et al., 2015), a list of differentially expressed genes was reduced from > 10,000 to four candidate genes. Similarly, Zhao et al. (2017) focused on the validation of 31 candidate genes from a pool of > 11,000 differentially expressed contigs in *Alopecurus aequalis*. Upon identification of candidate genes, functional analysis of the differentially expressed genes is necessary to confirm involvement in the mechanisms of resistance. Functional analysis can be achieved by performing knockout, knockdown, or upregulation of gene constructs in model plant organisms (however, see Mellado-Sánchez et al., 2020). Inherently, RNA-seq experiments are an exploratory approach, especially to design new hypotheses for a given phenotype, and should not be used as a stand-alone means to answer biological questions regarding NTSR.

Another strategy to identify NTSR is to look for signatures of selection in the weed genome. The idea behind this suite of techniques is to use population genomics approaches to identify loci under selection using a set of statistical tests. Because selection will shape the frequency of the alleles under selection, markers with unusual allele frequencies within and among populations may be compared using genetic markers. A number of approaches to acquire genetic markers have been used (Paris et al., 2010), with the bottom line to compare the distribution of marker data to a distribution of markers assumed to be under a neutral model of evolution. Although most types of markers may be used to perform such an analysis, single nucleotide polymorphisms (SNP’s) have been preferred as thousands of genome wide markers may be acquired with next-generation sequencing instruments. Genome-wide analysis provides the benefit of discovering new loci involved in the resistance traits. Many software programs have been developed to associate genotype with phenotype (reviewed by Hoban et al., 2016); however, a recent review of the outlier analysis usage between 2010 and 2016 (Ahrens et al., 2018) indicated ARLEQUIN was the most commonly program used for this purpose (Excoffier et al., 2005).

Outlier approaches are prone to a number of biases (e.g., false positives, confounded effects due to population structure, spatial correlation; reviewed by Hoban et al. (2016), therefore combining multiple approaches are typically beneficial to validate candidate loci. Examples of these approaches have been limited in the weed science literature, however are not absent. Kreiner et al. (2019) evaluated genetic differentiation using 100-kb sliding windows between resistant and susceptible *A. tuberculatus*, and found evidence that regions containing the EPSPS coding region were highly differentiated and likely involved in the resistance phenotype. Van Etten et al. (2020) studied eight populations of glyphosate-resistant and -susceptible *Ipomoea purpurea* from the Southeastern and Midwest United States that did not exhibit TSR. These authors adopted SNP outlier approaches to survival and resistance level data using and identified 42 to 83 loci (depending on the approach utilized) potentially involved in the glyphosate resistance trait. Following an exome resequencing step and outlier analysis, the authors were able to identify five genomic regions under positive selection that contained enriched genes in the cytochrome P450, ABC transporters, glycosyltransferases, and GST families. Although glyphosate metabolism has rarely been involved in the resistance mechanisms, more research is needed to confirm the involvement of metabolizing enzymes in *I*. *purpurea*. Similar approaches could be successful if implemented to uncover NTSR mechanisms in *Lolium* spp.

Genome-wide association studies (GWAS) rely on statistical models to find correlations between an observed phenotype and the genotype (reviewed by Leon et al., 2020). With high-throughput sequencing technologies, thousands to millions of SNP’s may be identified and can be used for association studies. These associations may be prone to high false positive rates if the statistical method chosen is not adequate to correct for confounding factors inherent from the study populations, such as population structure and unequal relatedness (Zhang et al., 2010). Several methods have been established to reduce false positive errors (Segura et al., 2012; Liu X. et al., 2016), and GWAS has been successfully implemented to identify the underlying genetic basis of traits in plants (Huang and Han, 2014). Although the availability of a reference genome may assist in the functional analysis of genomic regions, GWAS may be performed *de novo* (without the aid of a reference genome) (Voichek and Weigel, 2020). Limited weed science literature is available where GWAS was used to detect NTSR. Kreiner et al. (2020) conducted a GWAS in glyphosate-resistant *A. tuberculatus* that exhibited increased EPSPS duplication in the majority of the populations tested and, as expected, found that the genomic regions containing the EPSPS coding sequences were related to the resistance phenotype. The authors also found > 100 genes across the weed genome involved in the glyphosate resistance, and were identified as involved in stress-response and NTSR.

Regardless of the method employed to identify SNP’s throughout the weed genome, it is crucial that a thorough analysis is performed before the start of any experiment. An important consideration is the genome size. Analysis that rely on genome-wide SNP’s are typically performed with the assistance of restriction enzymes, which vary in the frequency that they cleave their recognition site. If a rate cutter restriction enzyme is chosen for a genome the size of *Lolium* spp. (approximately 2 Gb), it is very unlikely that the identified SNP’s will be physically linked (i.e., in linkage disequilibrium) with the causal mutation. Another consideration is the approach to construct the plant population that will be used for the analysis (reviewed by Morrell et al., 2012), which will also determine the likelihood of success in determining the genomic regions involved in the NTSR.

## Conclusion and Future Directions

*Lolium* spp. exhibit an astonishing potential to evolve herbicide resistance, likely due to its high genetic diversity and ability to exchange genetic material due to gene flow (Matzrafi et al., in press). *Lolium* spp. populations around the world have evolved NTSR to many herbicides. Because NTSR may be non-specific, populations may exhibit unknown herbicide resistance patterns, as resistance occurs to herbicides to which populations have never been exposed. NTSR poses a challenge to sustainable agricultural production systems, and is an ongoing issue that needs a collaborative approach to be minimized.

Non-target-site resistance research has elucidated fascinating aspects of how *Lolium* spp. evolve herbicide resistance, and adopted creative approaches to uncover the details of how plants manage to survive lethal doses of herbicides. Most of the efforts have been to describe the physiological and biochemical alterations that take place at the plant and cellular level (e.g., reduced herbicide translocation, herbicide metabolism). The underlying genetic bases of the phenotypes remain largely unknown. For instance, it is currently unknown which genes are involved in the vacuolar sequestration of paraquat in *L*. *multiflorum*. It is also unknown how these herbicide resistance genes arise in the populations (see modes of convergent adaptation in Lee and Coop, 2017). Information on the underlying genetic basis of the resistance mechanism has not only basic, but also applied applications. For instance, genetic markers may be developed to identify seed lots contaminated with herbicide resistant *Lolium* spp. seed, or field diagnostics to quickly identify herbicide susceptibility before growers treat an infested field. Policymakers may use information on how resistance genes arise in the population (i.e., gene flow, standing genetic variation, or new mutations; see Lee and Coop, 2017) to design regulations to prevent gene flow. Advancing our knowledge on NTSR resistance in *Lolium* spp. will require efforts of multidisciplinary teams that will likely include weed scientists, population geneticists, plant physiologists, biochemists, stakeholders, and funding agencies.

## Author Contributions

AKS, LB, CM-S, and CB wrote the manuscript. CM-S and CB conceptuaized the project. CB supervised the project. All authors contributed to the article and approved the submitted version.

## Conflict of Interest

The authors declare that the research was conducted in the absence of any commercial or financial relationships that could be construed as a potential conflict of interest.
